# Adventure Recreation in Blue Spaces and the Wellbeing of Young Polish Adults

**DOI:** 10.3390/ijerph20054472

**Published:** 2023-03-02

**Authors:** Piotr Próchniak, Agnieszka Próchniak

**Affiliations:** 1 Institute of Psychology, University of Szczecin, 70-453 Szczecin, Poland; 2 Department of Sociology, Pomeranian University, 76-200 Slupsk, Poland

**Keywords:** adventure recreation, wellbeing, blue spaces, young adults

## Abstract

The aim of this study was to assess the wellbeing of 248 young Polish adults between 18 and 26 years old (M = 22.35; SD = 2.20) involved in adventure blue space recreational activities. The adventure water recreational activities were measured by using a questionnaire specially designed for the purpose of this study. This questionnaire consisted of two subscales: adventure recreation associated with water risks and adventure recreation associated with weather risks. In turn, wellbeing was measured using six scales loaded in two factors: hedonic wellbeing and eudaimonic wellbeing. The regression analysis indicated that wellbeing (hedonic and eudaimonic) was positively predicted by adventure recreation associated with water risks. In turn, eudaimonic wellbeing was negatively predicted by adventure recreation associated with weather risks. Additionally, the cluster analysis revealed three distinct clusters of recreationists characterized by diverse results on the scales of adventure recreation dealing with water and weather risks: *soft adventurers* (low water risks/high weather risks), *hard adventurers* (high water risks/high water risks) and *avoiders* (low water risks/low weather risks). The hard adventurers had significantly higher means on hedonic wellbeing than that of the soft adventurers and the avoiders. Surprisingly, the soft adventurers had a significantly lower mean on eudaimonic wellbeing than that of the group of hard adventurers and the group avoiding risky activity in an aquatic environment.

## 1. Introduction

Blue spaces (the collective term for rivers, lakes, seas and oceans) are perceived as attractive places to spend free time and as a destination for holiday trips. Every year, millions of people relax in hotels, guest houses and campsites situated near a water environment. More and more people are taking p swimming, surfing, canoeing, scuba diving, cliff diving, sailing or kayaking. It is a way of expressing one’s own needs, desires or dreams.

### 1.1. Blue Spaces and Restoration

Several researches have reported on the role of exposure to natural environments in the promotion of wellbeing [1,2,3]. The literature suggests several possible psychological, social or physical benefits during activity in green or blue spaces [4]. One prominent framework is Attention Restoration Theory (ART), proposed by Stephen and Rachel Kaplan in the late 1980s [5]. The Kaplans distinguished between two forms of attention in a natural environment: voluntary attention and involuntary attention. Voluntary attention requires effort, self-control and direction. This leads to the exhaustion of attention resources and thus a reduction in quality of life. Its opposite is involuntary attention, which dominates in the natural environment. Involuntary attention does not require systematic effort, self-control or direction. Spending time in a natural environment has the capacity to restore attention and improve resistance to stressful life events [6].

Blue spaces in particular have an increased ability to promote psychological restoration relative to urban ones [7]. From a restorative perspective, blue spaces promote the regeneration of resources through mental detachment from the stress of the urban environment, fascination with the natural environment and commitment and internal motivation to achieve goals in the natural environment. Exposure to blue spaces can also help us improve attention [8].

Contact with blue spaces increases positive emotional states. For example, short walks surrounded by nature (e.g., a beach walk) can lead to a significant increase in positive mood [9]. Repeated contact with nature leads to better emotional functioning and greater satisfaction with life (Pearson et al., 2019) [10]. Moreover, living in close proximity to blue spaces increases the personal meaning of life, regardless of age [11]. Contact with nature promotes the reduction of stress, depression, internal tensions and anger [11,12,13,14]. People use blue spaces to be together with other individuals and enjoy social activities [15]. Blue space exposure is associated with increased time with family or friends [16]. Closer proximity to blue space promotes social interaction, neighbourhood attachment and social cohesion [17,18]. Contact with water is also important for powerful aesthetic and even transcendental experiences. The landscapes of lakes, seas and oceans are those that evoke admiration for the beauty and power of nature [19]. Contact with nature can also make people feel part of a larger project beyond their individual lives [20,21]. Exposure to blue spaces can promote the use of more effective coping strategies, can help meet the human need for authenticity, autonomy and competence, and may lead to the growth of positive emotions such as satisfaction and pleasure [16,22,23]. It is therefore no surprise that blue spaces can also promote different forms of recreation that are carried out in or on water.

### 1.2. Blue Spaces and Forms of Recreation

Recreation in blue spaces can be categorized into different forms. For example, we can distinguish primary and secondary recreational water activities. Primary recreational water activities are defined by the amount of water contact with different parts of the body or face. Examples of such primary activities are: bathing, water play by children, swimming, diving, surfing and windsurfing. Secondary recreational water activities have less contact with water (e.g., kayaking, sailing and fishing) [24].

Another categorization distinguishes between active and passive forms of water recreation. Passive recreation involves less physical effort or mental energy (e.g., sunbathing on beaches or wildlife observation) [25]. No special equipment or infrastructure is required for this type of recreation. The experience of pleasure, joy and the absence of stress is associated with this form of recreation. Passive forms of recreation have a minimal impact on the natural environment. In turn, active recreation, which entails direct participation, involves undertaking physical activity in contact with water (e.g., swimming, kayaking, cannoning, surfing, scuba-diving, sailing, cliff diving and water skiing). It is usually necessary to have special equipment in order to practise this type of leisure activity. Active recreation is performed for relaxation or for fun, but sometimes it is also performed to relieve stress.

The next categorization of recreation in blue spaces is linked to adventure forms [26,27]. It seems that swimming close to the shore on a beach patrolled by lifeguards, kayaking on a shallow, lowland river and wandering along coastal dunes are examples of soft adventure recreation in blue spaces. This type of recreation is usually less physically demanding and requires little experience from the participants [28]. The margin of error in safe recreation is large and the probability of an accident is very low. In turn, hard adventure recreation is riskier. However, diving at considerable depths, jumping from a high cliff into the water and surfing on high waves are examples of hard adventure recreation in blue spaces. Any error in hard recreation may prove fatal. These forms of activity are associated with the experience of very strong excitement, but also terror [26,29,30,31].

Hard adventures require belief in one’s capabilities because the physical demands are very high [32,33]. However, self-efficacy can promote underestimation of the levels of risk in outdoor recreation. Participants of outdoor adventure often report not experiencing such activity as very risky, but objective statistics suggest a high probability of injury or even death [34].

Weather can be one of the most universal aspects of adventure in water sports [35,36]. Recreationists might experience strong winds, heavy rain or cold temperature of air and water. These weather conditions can be associated with significant mental and physical strain on explorers of blue spaces [37]. In this way, in certain specific situations harsh weather combined with water risks can be the spectre of a hard adventure in blue spaces. On the other hand, if recreation is practiced in difficult weather conditions but in a relatively safe water environment, then we can say that it is a soft adventure (e.g., a walk along the beach during rain or strong wind) [38].

Young adults take more hard adventures than any other age group [39,40,41,42]. The increase in the popularity of risky outdoor recreation in this group of people may be a sign of modern times. In the opinion of Puchan, young people are looking for risk because it can be associated with experiencing new sensations and realization of their own needs and values [43]. Hard adventure recreations might also be useful in enhancing physical activity and promoting psychological wellbeing in young people [44].

### 1.3. Recreation in Blue Spaces and Wellbeing

Both soft adventure and hard adventure recreations can have positive consequences for subjective wellbeing; however, this construct is not easy to define. In the literature, a lot of definitions exist for wellbeing. Researchers propose different dimensions of subjective wellbeing, such as happiness, life satisfaction, meaning in life, flow, flourishing, vitality, hope, optimism, positive affect and lack of a negative affect [45,46,47,48,49].

Martin Seligman (2011) proposed a model of wellbeing which includes the following components: positive emotions (P—e.g., joy, recognition, comfort, inspiration, curiosity and hope), engagement (E—e.g., focusing on the activity performed and observing what happens around me), relationships (R—e.g., cooperation with others and feeling supported by others), meaning (M—e.g., sense of meaning and sense of activity) and accomplishments/achievements (A—e.g., mastery, competence and the concept of having the passion to attain goals). It is often referred to as the PERMA model. In turn, Newman et al. proposed a conceptual model for understanding the interplay between leisure and subjective wellbeing. It encompasses five psychological mechanisms as follows: detachment-recovery, autonomy, mastery, meaning and affiliation (DRAMMA) [50].

Wellbeing can be classified as hedonic and eudaimonic wellbeing. Hedonic wellbeing is defined by a high level of positive affect and a low level of negative affect [51]. The experience of pleasure, joy or relaxation is associated with this form of wellbeing. In turn, eudaimonic wellbeing includes a sense of meaning and purpose in life or investment of significant effort in the pursuit of excellence, low frequencies of negative affect and a global cognitive evaluation of life as satisfying [52]. From the perspective of activity theories which assume that activity is the basic form for any living organism, hedonic wellbeing’s role is to regulate emotional stability according to the principles of homeostasis. In turn, the role of eudemonic wellbeing is to regulate change [53].

Recreation in blue spaces is linked to different aspects of wellbeing. For example, a study conducted by A. Morgan and her co-workers indicated that scuba diving decreases levels of anxiety or depression and improves social functioning [54]. In a study conducted by Carreño and his co-workers, scuba diving activities had positive effects on human mental health [55]. Participants of the Surf-Salva Camp 2016 indicated that surfing had a number of positive effects as follows: exploration, effort and perseverance, problem-solving, time-management, social competencies, interpersonal relationships and emotional regulation [56]. In a study by Rocher et al., children and adolescents from the School Nautical Activities project in Portugal who took part in water recreations (e.g., surfing, rowing, sailing and canoeing) declared benefits in the physical, mental, educational and social dimensions [57]. Participants of a study by Fredrickson and Anderson described the waterscape of canoe country as a source of spiritual inspiration [20].

The above studies were conducted on rather safe recreation in blue spaces. Researchers have also investigated the importance of hard adventure recreation in blue spaces for mental health. Recreationists who undertake extreme or risky recreations in blue spaces (big wave surfing, waterfall kayaking) talk about a sense of freedom, a full sense of their lives or a sense of connection with nature [39]. Jones and his colleagues found that canoeists overcoming difficult mountain rivers experience deep satisfaction with life more often than anxiety, boredom or apathy [58]. For surfers, the opportunity to confront high ocean waves is a source of exciting positive emotions and deep satisfaction [59].

Research on the relationship between hard adventure recreation and wellbeing is usually conducted in the form of interviews with adventure participants. However, it is difficult to state unequivocally whether undertaking risky recreation actually promotes the increase in wellbeing in these groups of people or whether these people only experience happiness retrospectively returning to the memories of the risk-taking period [32,60].

The above studies most often investigated the consequences of outdoor recreation for wellbeing. In other words, researchers aimed to determine how wellbeing changes when in contact with the natural environment. On the other hand, there is a lack of research on the everyday wellbeing of people undertaking adventure recreation in blue spaces. Thus, the aim of this article is to analyse relation between different aspects of adventure recreation (adventure recreation associated with water risks and adventure recreation associated with weather risks) in blue spaces and wellbeing. Another goal of this study was to distinguish types of water recreationists depending on the type of adventures they undertake in the blue spaces.

## 2. Method

### 2.1. Participants

A total of 248 participants (98 women and 150 men) took part in this research. The average age of the surveyed people was M = 22.35; SD = 2.20. Most of the respondents (68% of respondents) lived in cities, and the others (32% of respondents) lived in the countryside. All of the respondents lived in the Pomeranian Region (Poland) near the Baltic Sea. There are also numerous rivers, lakes, two coastal national parks (Slowinski National Park and Wolinski National Park) and thirteen local landscape parks in this region. All participants had at least a secondary level of education.

### 2.2. Procedure

Ethics approval was obtained (from the University of Szczecin Institutional Review Board) before we began recruiting participants. Water recreationists were invited to participate. The researchers asked the recreationists whether they liked to spend their free time by the water. Those who liked to spend their free time by water and provided consent to participate in the study received a set of questionnaires to be completed individually. The questionnaires were available in the Polish language. Each person was informed about the purpose of the study and was assured that the results of the study would be used only for scientific purposes. The participants needed about 20 min to complete the set of questionnaires. They filled in the questionnaires individually at home and returned them to the authors. Data collection took place between March and April 2022.

All of the participants were selected on the basis of the following criteria:

(a) they were young persons between 18 and 26 years old (e.g., younger persons may be more willing to take risks) [61,62];
(b) participants practiced recreation in an aquatic environment for at least 7 days per year;
(c) participants had to be involved in aquatic recreation at the Baltic Sea;
(d) participants were motivated to participate in the research.

From 276 interested participants, 28 did not meet the eligibility criteria. The exclusion criteria were: (a) age below 18 or above 26 years old and (b) incidental recreation in the blue spaces.

### 2.3. Measures

#### 2.3.1. The Adventure Recreation in Blue Spaces Questionnaire (ARBSQ)

The Adventure Recreation in Blue Spaces Questionnaire was specifically designed for this study. We shortly present the phases of construction of The Adventure Recreation in Blue Spaces Questionnaire.

The research related to leisure indicates that adventure in blue spaces includes forms associated with a higher possibility of accidents, for example jumping from a high cliff or diving underwater without the use of breathing apparatus. In addition, strong winds, rain, cold air and cold water can place a significant mental and physical strain on water recreationists. Therefore, we distinguished two aspects of adventure recreation in blue spaces: adventure recreation associated with water risks and adventure recreation associated with weather risks.

Using these assumptions, we generated eleven statements about adventure activities associated with water risks and ten statements about adventure activities associated with risky weather in aquatic environments.

The statements of the questionnaire did not include recreational activities that require spending large amounts of money on the purchase of equipment necessary to undertake the recreation (e.g., purchase of a boat, kayak or surfboard). As a result, it was possible to invite for research people who liked contact with aquatic environments regardless of their financial circumstances.

To assess the quality of the items, experts (who were specialists in environmental psychology) were asked to use a 5-point Likert-type scale (very poor, poor, fair, good, very good) to independently determine the extent to which the initial pool of items reflected adventure recreation associated with water risks. Using the same scale, experts evaluated the extent to which the initial pool of items reflected adventure recreation associated with weather risks. Items were retained if the average was 4.0 or higher.

This pre-selection process reduced the number of statements to fifteen (eight items for adventure recreation associated with water risks and seven items for weather risks). The list of fifteen items was administered to the first 253 sample respondents (123 women and 130 men) who practiced recreation in blue spaces (M = 22.40 yr; SD = 2.30).

Data obtained from this sample were examined by exploratory factor analysis—EFA. Prior to factor extraction, the Kaiser–Meyer–Olkin (KMO) measure of sampling adequacy and Bartlett’s Test of Sphericity (BTS) were applied to the data: KMO = 0.864, BTS = χ^2^ (105) = 1720.79, *p* < 0.0001. Exploratory factor analysis using the principal component analysis (together with Parallel Analysis) of the statements of the Adventure Recreation in Blue Spaces Questionnaire resulted in two factors. The first factor contained 5 items of recreation connected with water risks, and the second factor consisted of 5 items of water recreation connected with weather risks. Examples of risky recreation are the following statements:

*I like to jump into water from a steep slope, I like diving underwater without the use of breathing apparatus, I like to swim far from the shore*.

Examples of adventure recreation associated with weather risks are the following items:

*I like running on the beach in stormy weather, I like struggling with the wind during practicing water sports (See: Appendix A* *)*

Based on the results obtained in the EFA, confirmatory factor analysis (CFA) was conducted on scale scores in the second group of 233 participants (95 women and 138 men) (M = 21.20 lat; SD = 2.60). A confirmatory factor analysis confirmed a two-factor solution (GFI = 0.946; AGFI = 0.913, CFI = 0.952, RMSEA = 0.0497; χ2 (34) = 68.29; *p* = 0.01). The *Adventure Recreation in Blue Spaces Questionnaire* is a reliable instrument: the reliability of the subscale of adventure recreation associated with water risks is *Cronbach’s α* = 0.84. Similarly, the reliability of the scale for adventure recreation associated with weather is *Cronbach’s α* = 0.72.

Convergent validity of the Adventure Recreation in Blue Spaces Questionnaire was assessed by examining the relationship between *ARBSQ* and other similar constructs, as: adventure seeking scale [63], and sensation seeking scale [64]. Both scales of The Adventure Recreation in Blue Spaces Questionnaire correlated with similar constructs (but adventure recreation associated water risks correlated higher with these constructs than the second subscale did).

The authors also tested how the scale functions among people practicing high (kitesurfing) and low risk (a shallow river kayaking) outdoor activities (predicted validity). The kitesurfers scored higher on the adventure recreation scales than the kayakers group.

#### 2.3.2. Oxford Happiness Questionnaire (OHQ) [65]

The Oxford Happiness Questionnaire (OHQ) was originally a 29-item scale with all items included in one factor. The Polish version of the OHQ consists of two subscales: general satisfaction with life (*Cronbach’s α* = 0.88) and meaning of sense and control of life (*Cronbach’s α* = 0.82) (Polish adaptation: Ref. [66]. Respondents indicate how much they agree or disagree with each statement, according to a Likert scale from 1 (strongly disagree) to 6 (strongly agree).

#### 2.3.3. Meaning of Life Questionnaire (MLQ) [67]

The MLQ is originally a 9-item scale with two subscales: presence of meaning in life (*Cronbach’s α* = 0.86) and search for meaning in life (*Cronbach’s α* = 0.72). (Polish adaptation: Ref. [68]. Respondents answer each item on a 7-point Likert-type scale ranging from 1 (absolutely true) to 7 (absolutely untrue).

#### 2.3.4. Positive and Negative Affect Schedule (THE PANAS) [69]

The PANAS was created to provide brief measures of positive affect (PA) and negative affect (NA). The correlation coefficients were 0.73 for positive affect and 0.90 for negative affect. (Polish adaptation: Ref. [70]. Responses are rated on a 5-point Likert scale ranging from 1 (very slightly or never) to 5 (very much).

#### 2.3.5. The Temporal Satisfaction with Life Scale

The Temporal Satisfaction with Life Scale [71], is a 15-item self-reported instrument intended to diagnose the respondent’s past (Cronbach’s *a* = 0.81), present (Cronbach’s *a* = 0.79) and future life satisfaction (Cronbach’s *a* = 0.81). Responses are rated on a seven-point Likert scale ranging from 1 (absolutely untrue) to 7 (absolutely true). The Polish adaptation: Ref. [72].

#### 2.3.6. Ego Resiliency Scale [73]

The Ego Resiliency Scale consists of 14 items. It measures resiliency in different situations. The scale has a satisfactory internal consistency (*Cronbach’s α* = 0.78) (Polish adaptation: Kaczmarek, 2011) [74]. Participants respond to each item using a 4-point scale ranging from 1 (does not apply at all) to 4 (applies very strongly).

#### 2.3.7. Life Orientation Test Revised (LOT-R) [75]

The Life Orientation Test Revised (LOT-R) is a 10-item unidimensional scale that was constructed to assess individual differences in generalized optimism. The coefficient alpha in reliability in the Polish version for the Life Orientation Test was: *Cronbach’s α =* 0.73 (Juczyński, 2001) [76]. Responses are rated on a 5-point Likert scale ranging from 0 (strongly disagree) to 4 (strongly agree).

All tests were performed using Statistica 13.0.

## 3. Results

Table 1 presents basic statistics for analysed variables.

In the next step, the correlations between two subscales of the adventure recreation in blue spaces (weather risk and water risk) and the eleven scales of wellbeing were conducted (See Table 2).

The adventure recreation associated with water risks subscale correlated positively with positive affect and resilience. The adventure recreation associated with weather risks subscale correlated only with positive affect.

In next step, a factor analysis of the wellbeing scales was performed to discover the main factors of wellbeing in the sample of participants. Therefore, an exploratory factor analysis (EFA) was conducted for the wellbeing scales. The data were suitable for an EFA (KMO value of 0.874, significant Bartlett’s Test of Sphericity, *χ*^2^(45) = 1478.005, *p* < 0.001). The principal components method was chosen [77]. The results of the EFA are presented in Table 3.

The exploratory factor analysis using the principal components method indicated a two-factor solution on inspecting the scree plot. The Parallel Analysis also confirmed a two-factor solution [78].

The first factor accounted for 52% of the variance (subcomponents: satisfaction with life, meaning of sense and control of life, presence of meaning in life, satisfaction with time perspectives and negative affect). It seems that this factor relates to eudaimonic wellbeing. The second factor accounted for 12% of the variance (subcomponents: the search for meaning in life, positive affect, resiliency and optimism). This factor relates to hedonic wellbeing.

In the next step, regression analysis was used. Regression analysis is a tool to establish a relationship between two variables. One of these variables is called the independent variable (predictor). The other variable is called the dependent variable, whose value is derived from the independent variable. The two aspects of adventure recreation were treated as independent variables and two factors of wellbeing (hedonic and eudaimonic) as dependent ones.

Table 4 presents the regression correlations between the adventure recreation subscales and wellbeing factors.

Hedonic wellbeing was predicted by adventure associated with water risks. In turn, eudaimonic wellbeing was predicted positively by adventure associated with water risks and it was predicted negatively by adventure associated with weather risks.

In the another stage of the study, clustering analysis for the subscales of adventure recreational activities was used (K-means clustering subscales and method). The main goal of a cluster analysis is to group respondents into clusters. The respondents in a cluster should be similar to one another and be different from the respondents in the other clusters. The clustering analysis was used in this study to extract the basic clusters for individuals who undertake risky and safe water recreation. In other words, we were looking for respondents who had similar scores on the risky and safe recreation subscales within a cluster and had different scores from the respondents grouped in other clusters.

We tested different numbers of clusters. The K-means cluster method showed that the cluster model with the best fit was the three cluster model. In this model, the variance between the groups was higher than the variance within the groups for the safe and risky recreational activities subscales simultaneously (higher variance between groups than variance within any single group is an important criterion in extracting clusters) (See Table 5).

**Figure 1:**
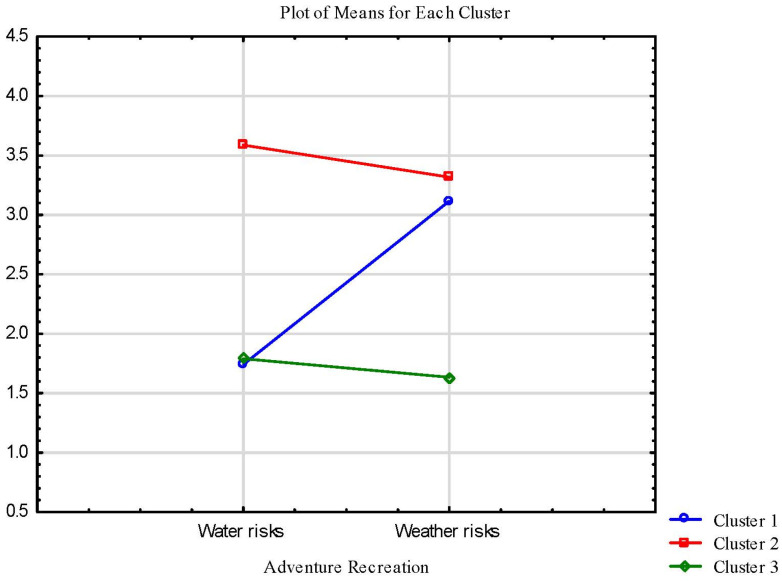
Clusters of adventure recreation in blue spaces; results of cluster analysis.

The first cluster comprised 54 respondents who scored low on the water risky activities subscale and high on the weather risks one (*soft adventurers*). The second cluster contained 105 individuals who had high scores on both recreational activities scales (e.g., a high score on the hard adventure associated with water risks subscale and a high score on the hard adventure associated with weather risks subscale) (*hard adventurers*). The last cluster was composed of 89 respondents who received low scores on both scales for hard adventure activities (*avoiders*) (See Figure 1).

In the final step, we compared the scores on wellbeing factors in the three clusters of respondents (See Table 6).

The group that practised recreations associated with water and weather risks had significantly higher means on hedonic wellbeing than that of the group that undertook adventure recreation only associated with weather risks and the group avoiding risky activity in blue spaces. Additionally, the group of adventurers who liked weather risks had a significantly lower mean on the eudaimonic wellbeing factor than the group of adventurers who liked water risks and the group avoiding risky activity in an aquatic environment.

## 4. Discussion

Scientists are interested in determinants of mental health [49,79]. One such variable is leisure time activity [50,80]. Physical activity in free time, including outdoor recreation, is especially associated with an increase in personal wellbeing. Recreation has a positive effect on various physiological, affective and social wellbeing processes [2,4].

Our own research results suggest that only adventure recreation associated with water risks in blue spaces is related to mental health. The adventure recreation associated with water risks has a positive relationship with hedonic and eudaimonic wellbeing. This type of recreation can enhance sense of life, satisfaction, power or positive emotions. In this context, our results confirm previous studies related to the positive consequences of risk-taking in recreation for wellbeing [58,59].

The positive correlations between adventure associated with water risks and wellbeing can be explained by psychological perspectives referring to the different models of wellbeing. Both the PERMA and DRAMMA models emphasize the importance of achievements (PERMA model) or mastery (DRAMMA model) in creating wellbeing [48,50,81]. Hard adventure in blue spaces can promote these features; hence, its relationship with wellbeing seems obvious [17,18].

Of course, we must remember that correlations between adventure recreation associated with water risks and wellbeing are rather low and could be considered more as a trend. It means that the path to seeking mental health by engaging in risky activities in close contact with water is not a much more effective path than undertaking safe recreation. In other words—it seems that wellbeing can be increased without voluntarily engaging in dangerous water activities.

For hedonic wellbeing, undertaking adventure recreation associated with weather risks in blue spaces does not matter. In turn, adventure associated with weather risks was negatively related to eudaimonic wellbeing.

Why does adventure recreation associated with weather risks not promote satisfaction with life? It can be interpreted from the Attention Restoration Theory proposed by Kaplan [6]. Kaplan claims that activity in the natural environment must not contain any unexpected or surprising elements [6]. Strongly stimulating, dangerous, new or difficult activity requires large amounts of attention, which leads to exhaustion. Harsh weather in blue spaces can include strong wind, rain or cold water and air, and probably engages the processes of voluntary attention, which is not conducive to life satisfaction.

The goal of our study was also to compare the subjective wellbeing of individuals who engaged in different forms of adventure recreation in blue spaces. The group that practised recreations associated with water risks had significantly higher means on wellbeing (hedonic and eudaimonic factors) than that of the group that undertook adventure recreation associated with weather risks. Additionally, the group of adventurers who liked water risks had a significantly higher mean on the hedonic wellbeing factor than that of the group avoiding risky activity in an aquatic environment. Interestingly, the group of adventurers who liked weather risks had a significantly lower mean on the eudaimonic wellbeing factor than that of the group avoiding risky activity in blue spaces.

The results can be interpreted in the light of the biological base of physical activity. Many studies indicate that risky physical activity releases hormones such as endorphins and serotonin [82]. These hormones improve positive mood or energy levels and decrease anxiety [4,83]. Moreover, physical exercise pumps blood to the brain, which can help people to improve thinking, problem solving, attention or learning; hence, its relationship with mental health and positive functioning seems obvious [84,85]. Hard adventurers who like water risks can activate these biological process more often and therefore they can have higher scores on emotional wellbeing when compared with other recreationists.

It is difficult to explain that the group of adventurers who liked weather risks had a significantly lower mean on the eudaimonic wellbeing factor than that of the group avoiding risky activity in blue spaces. Research shows that harsh weather conditions can alter a person’s physiological functioning: severe weather can have a huge impact on body temperature, blood pressure or the endocrine system. The result can be hypothermia. Adverse weather can also affect psychological functioning—perception, decision making, rational thinking, fear or aggression—which can lead to psychological disorders such as depression or generalised anxiety disorder [86,87,88,89,90,91,92].

Considering the above (strong winds, cold water, heavy rain or fog) can lead to the exhaustion of personal resources during practising outdoor recreation and, thus, a reduction in quality of life (wellbeing). Another explanation is the following: adventurers who like weather risks may have a tendency to become depressed (lower wellbeing), but this hypothesis requires further investigation.

## 5. Applications of This Study

The research results may have practical benefits for education. It turns out that taking risks in blue spaces was associated with subjective wellbeing.

This result can be an important argument for getting involved in adventure recreation, especially for people who are reluctant to take on challenges in the natural environment. It can also be a valuable insight for people running adventure therapy and survival schools.

Not all forms of blue space adventure recreation promote wellbeing—activity in difficult weather conditions can actually reduce an individual’s wellbeing. Information on the influence of weather on adventure recreation can be a source of reflection, especially for young adults, who are statistically more likely to attempt dangerous challenges. Of course, it is difficult to assess the degree to which this information will change young people’s future behaviour including their selection of blue space activities. Our future behaviours are not only influenced by external forces, but also by internal ones, such as biologically determined personality traits and cognitive processes [93].

## 6. Limitations of the Study

An important limitation of the study is that it only capture the experiences of young people. This fact limits the generalizability of the results. In future research, it will be important to assess not only young people, but other groups of adults. In this study, information on the gender of the participants was not collected.

Future research should also take into account gender and control for it in the analyses. Previous research indicates that variables of age and gender can play an important role in practising outdoor recreation [94,95]. Under this study, only some of the variables that contribute to wellbeing were subjected to analysis. Future research might encompass some of the concepts of wellbeing which were not incorporated, such as flow [49].

The data were collected in Poland. Several items can concern specific local conditions which are characteristic for Poland. It might be difficult for people from other geographical regions to respond to these statements.

An important limitation of the present study is that some correlations and differences between groups are low and could be considered more as a trend. Future studies should collect data from larger samples of participants in each type of participation category.

## 7. Conclusions

Nature has a lot to offer. From a biological point of view, it provides food, water and is the source of many medicines. The psychological benefits are also very important. It is the opportunity to experience the beauty of nature and the opportunity to learn, discover and explore nature. Nature also offers emotional benefits such as the feeling of joy, relaxation and reduction of stress. The results of this study indicate that risky recreation in close contact with water can promote wellbeing, but this promotion is rather weak.

Exploration of the natural environment also gives us an opportunity to experience thrill and excitement, fear of death and peak experience. Risk-taking in recreation satisfies the need for stimulation and willpower. However, the path to seeking satisfaction in life by engaging in dangerous activities in close contact with nature is not an effective path. This knowledge should be passed on, especially to young people who risk their own lives in the name of short-lived, positive emotions.

## Figures and Tables

**Table 1:** Number of participants, medium, minimum, maximum standard deviation, skewness and kurtosis for analysed variables.

	N	M	Minimum	Maximum	SD	Skewness	Kurtosis
Water Risks	248	2.65	1.00	5.00	1.14	0.20	−1.00
Weather Risks	248	2.59	1.00	5.00	0.94	0.21	−0.50
General Satisfaction of Life	248	3.58	1.00	5.00	0.69	−0.69	1.07
Meaning of Sense and Control of Life	248	3.32	1.00	5.00	0.82	−0.25	−0.07
Presence of Meaning in Life	248	4.35	1.00	7.00	1.44	−0.41	−0.34
Search for Meaning in Life	248	5.28	1.00	7.00	1.21	−1.01	1.42
Positive Affect	248	3.51	1.00	5.00	0.67	−0.23	0.41
Negative Affect	248	2.84	1.00	5.00	0.78	0.31	−0.20
Past Satisfaction	248	4.22	1.00	7.00	1.34	−0.20	−0.29
Present Satisfaction	248	4.74	1.00	7.00	1.33	−0.56	−0.03
Future Satisfaction	248	5.25	1.00	7.00	1.26	−0.85	0.73
Resilience	248	3.01	1.00	4.00	0.49	−0.38	0.34
Optimism	248	2.74	1.00	4.00	0.56	−0.19	−0.26

**Table 2:** Correlations between the adventure recreational activities and wellbeing.

Variables	Water Risks	Weather Risks
General Satisfaction with Life	0.12	−0.01
Meaning of Sense and Control of Life	0.02	−0.08
Presence of Meaning in Life	0.01	−0.07
Search for Meaning in Life	0.09	−0.03
Positive Affect	0.25 *	0.12 *
Negative Affect	−0.01	0.02
Past Satisfaction with Life	0.07	−0.01
Present Satisfaction with Life	0.06	−0.04
Future Satisfaction with Life	0.08	−0.09
Resiliency	0.20 *	0.12
Optimism	0.11	−0.01

* *p* < 0.05.

**Table 3:** Exploratory factor analysis of the wellbeing scales.

Variable	Factor 1	Factor 2
1. Satisfaction with Life	0.66	
2. Meaning of Sense and Control of Life	0.84	
3. Presence of Meaning of Life	0.58	
4. Search for Meaning in Life		0.70
5. Positive Affect		0.72
6. Negative Affect	−0.071	
7. Past Satisfaction with Life	0.69	
8. Present Satisfaction with Life	0.69	
9. Future Satisfaction with Life	0.56	
10. Resiliency		0.75
11. Optimism		0.60
Eigenvalues	5.72	1.04

**Table 4:** Wellbeing and adventure recreation. The results of multiple linear regression.

Variables	Hedonic Wellbeing		
	β	*t* (245)	*p*
Adventure associated with water risks	0.23	3.36	0.01
Adventure associated with weather risks	−0.07	−1.00	ns.
R^2^ = 0.04; F (2,245) = 5.88; *p* < 0.05			
	**Eudaimonic Wellbeing**		
	**β**	***t*** **(245)**	***p***
Adventure associated with water risks	0.14	2.06	0.05
Adventure associated with weather risks	−0.14	−1.97	0.05
R^2^ = 0.02; F (2,245) = 2.77; *p* < 0.05			

**Table 5:** Variance within and between groups for hard adventure recreation; the results of clustering analysis.

Model	Variable	Variance Between Groups	df	Variance Within Group	df	F	*p*
Two Clusters	Water risks	187.19	1	99.05	246	464.89	0.01
	Weather risks	92.55	1	149.35	246	152.43	0.01
Three Clusters	Water risks	199.34	2	86.90	245	280.98	0.01
	Weather risks	150.52	2	91.38	245	201.77	0.01

**Table 6:** Comparisons of respondents in three clusters on the wellbeing factors. The results of one-way ANOVA.

Factors of Wellbeing	Cluster Soft Adventurers (a)		Cluster 2 Hard Adventurers (b)		Custer 3 Avoiders (c)		F
	M	SD	M	SD	M	SD	
The Hedonic Wellbeing	3.46	0.59	3.76	0.53	3.60	0.52	5.51 a − b **; b − c *
The Eudaimonic Wellbeing	3.77	0.75	4.12	0.71	4.11	0.69	4.82 a − b **; a − c **

* *p* < 0.05. ** *p* < 0.01. a. Low water risks; high weather risks. b. High water risks; high weather risks. c. Low water risks; low weather risks.

## Data Availability

The datasets used during this study are available from the corresponding author.

## Appendix A

The Adventure Recreation in Blue Spaces Scale

I like to swim far from the shore;

I like to continue recreation in blue spaces even when it is cold;

I like diving underwater without the use of a breathing apparatus;

I like water recreation even on rainy days;

I like to jump into water from a steep slope;

I like running on the beach in stormy weather;

I like to check how long I can stay underwater;

I like struggling with the wind during practicing water sports;

I like to swim in an unguarded and previously unknown body of water;

I like to perform water recreation even on very cloudy days.
